# Ethylene Suppresses Abscisic Acid, Modulates Antioxidant System to Counteract Arsenic-Inhibited Photosynthetic Performance in the Presence of Selenium in Mustard

**DOI:** 10.3389/fpls.2022.852704

**Published:** 2022-05-16

**Authors:** Zebus Sehar, Noushina Iqbal, Mehar Fatma, Bilal A. Rather, Mohammed Albaqami, Nafees A. Khan

**Affiliations:** ^1^Plant Physiology and Biochemistry Laboratory, Department of Botany, Aligarh Muslim University, Aligarh, India; ^2^Department of Botany, Jamia Hamdard, New Delhi, India; ^3^Department of Biology, Faculty of Applied Science, Umm Al-Qura University, Makkah, Saudi Arabia

**Keywords:** abscisic acid, antioxidant, arsenic, ethylene, selenium

## Abstract

Arsenic (As) stress provokes various toxic effects in plants that disturbs its photosynthetic potential and hampers growth. Ethylene and selenium (Se) have shown regulatory interaction in plants for metal tolerance; however, their synergism in As tolerance through modification of the antioxidant enzymes and hormone biosynthesis needs further elaboration. With this in view, we investigated the impact of ethylene and Se in the protection of photosynthetic performance against As stress in mustard (*Brassica juncea* L.). Supplementation with ethephon (2-chloroethylphosphonic acid; ethylene source) and/or Se allayed the negative impact of As-induced toxicity by limiting As content in leaves, enhancing the antioxidant defense system, and decreasing the accumulation of abscisic acid (ABA). Ethylene plus Se more prominently regulated stomatal behavior, improved photosynthetic capacity, and mitigated As-induced effects. Ethephon in the presence of Se decreased stress ethylene formation and ABA accumulation under As stress, resulting in improved photosynthesis and growth through enhanced reduced glutathione (GSH) synthesis, which in turn reduced the oxidative stress. In both As-stressed and non-stressed plants treated with ethylene action inhibitor, norbornadiene, resulted in increased ABA and oxidative stress with reduced photosynthetic activity by downregulating expression of ascorbate peroxidase and glutathione reductase, suggesting the involvement of ethylene in the reversal of As-induced toxicity. These findings suggest that ethephon and Se induce regulatory interaction between ethylene, ABA accumulation, and GSH metabolism through regulating the activity and expression of antioxidant enzymes. Thus, in an economically important crop (mustard), the severity of As stress could be reduced through the supplementation of both ethylene and Se that coordinate for maximum stress alleviation.

## Introduction

Arsenic (As) is considered a toxic metalloid of global concern on account of increasing water contamination in many parts of the world, particularly Bangladesh, China, India, and Taiwan (Srivastava et al., 2016; Murugaiyan et al., 2019; Abedi and Mojiri, 2020; Bali and Sidhu, 2021). Numerous factors including geochemical, microbial, and human activities contribute significantly to As mobilization in the environment. In plants, As absorbed from the soil as arsenate (As V) or arsenite (As III) enhances generation of reactive oxygen species (ROS) that intensify oxidative stress, such as superoxide radicals, singlet oxygen, hydroxyl radicals, and hydrogen peroxide (H_2_O_2_) (Tripathi et al., 2007; Khan et al., 2021a; Nahar et al., 2022), resulting in damage to macromolecules and the inhibition of photosynthetic performance, growth, and productivity (Rahman et al., 2015; Farnese et al., 2017; Singh et al., 2017; Khan et al., 2021a,b; Asgher et al., 2022). Several studies have documented plant sensitivity to As in contaminated soil or in an artificial setting, and reported that it interrupts plant developmental processes and antioxidant system (Srivastava et al., 2016; Asgher et al., 2021; Bali and Sidhu, 2021).

Strategies for As mitigation include the study of As sequestration in roots during As stress and the enhancement of the antioxidant system. Asgher et al. (2022) found supplementation of 10-μM L-glutamic acid reduced As stress on photosynthetic and growth attributes through enhancement of antioxidant and proline metabolism. The modern approach to enhancing plant adaptability to heavy metal stress is to address the crosstalk between phytohormones and mineral nutrients, enabling plants to synthesize reduced sulfur (S) compounds for detoxification of metal-induced ROS. The gaseous hormone ethylene has been shown to regulate every aspect of plant growth and production in both ideal and stressful conditions (Iqbal et al., 2011; Riyazuddin et al., 2020; Fatma et al., 2021; Sehar et al., 2021). The action of ethylene is determined by its cell concentration and the response of plant to the hormone (Arraes et al., 2015; Sun et al., 2016). Ethylene plays a significant role in stress acclimation and mediates a diverse array of signaling processes under abiotic stress conditions. Broadly, ethylene controls plant responses to diverse environmental conditions by increasing antioxidant enzyme levels (Arraes et al., 2015; Riyazuddin et al., 2020). Dietz et al. (2010) showed that ethylene response factors (ERF) proteins mediate and integrate hormonal and redox signaling pathways during abiotic stress. The increase in antioxidant enzymes activity and reduced glutathione (GSH) content is the central defense tolerance strategy adapted by plants exposed to stress. In transgenic *N. tabacum* plants the enhanced GSH content and *ACO* ge*ne* expression and *ERF* gene signaling suggest the role of ethylene in GSH production and stress tolerance (Ghanta et al., 2014). Under salt stress, ethylene-induced GSH production and increased the expression of the photosystem II (PSII) genes to protect PSII activity and photosynthesis (Sehar et al., 2021). Plant hormone networks are complex and might influence each other's synthesis, degradation, or sensitivity. Ethylene works antagonistically with abscisic acid (ABA) and regulates guard cell signaling (Nazareno and Hernandez, 2017). Fatma et al. (2021) showed both ethephon and S influenced ABA content and stomatal regulation in mustard under salt stress. Ethylene signaling mutant (*etr1, ein2, ein3*) and ABA pathway mutants (*aba1, aba2, abi1, abi2*) were found to antagonistically regulate defense and stress–responsive genes expression under biotic and abiotic stress responses (Ghassemian et al., 2000; Yanagisawa et al., 2003; Beguerisse-Diaz et al., 2012). Ethylene is reported to induce stomatal opening in some species and inhibited ABA-induced stomatal closure (Tanaka et al., 2005; Benlloch-González et al., 2010). Wang and Song (2008) found that ethylene receptor ETR1 plays an important role in stomatal regulation. Besides acting as a receptor for ethylene perception it also mediates H_2_O_2_ signaling. The H_2_O_2_ signaling falls within the ABA response pathway. Thus, when ETR1 binds to ethylene, it inhibits the alternative signal of the presence of H_2_O_2_, reducing the stomatal response to ABA. Moreover, crosstalk between ethylene, NO, and salicylic acid has been suggested for As toxicity in rice (Khan et al., 2021a).

Selenium (Se) is well-known as a beneficial element, functioning in the control of growth and development under normal and stressful conditions at lower concentration (Khan et al., 2015; Hasanuzzaman et al., 2022). Selenium enhances the metals/metalloids toxicity tolerance in plants (Hasanuzzaman et al., 2020a,b, 2022; Zhang et al., 2020). It has been shown to detoxify ROS through upregulating the activity of antioxidant enzymes (Hasanuzzaman and Fujita, 2011; Srivastava et al., 2016; Hasanuzzaman et al., 2020a,b), leading to the protection of photosynthetic and growth capabilities (Khan et al., 2015). Supplying exogenous Se to *Brassica napus* improves enzymatic and non-enzymatic antioxidant processes and increases GSH content, enhancing plant immunity to Cd-induced oxidative harm (Hasanuzzaman et al., 2012). Selenium, on the other hand, works as an antioxidant only at low concentrations, while at higher concentrations, it acts as a pro-oxidant, causing an increase in ROS and lipid peroxidation (Chauhan et al., 2019; Hasanuzzaman et al., 2020b). In addition, Panda et al. (2017) found efficient translocation of As to shoot tissue by regulating cellular redox homeostasis and antioxidant defense system. Similarly, Khan et al. (2015) found that in *Triticum aestivum*, the actions of Se and S are comparable in reversing Cd-induced oxidative stress, the mechanism of which prominently involved control of ethylene production, S-assimilation, and GSH production. Selenium enhanced antioxidant enzymes activities that reduced the levels of oxidative stress markers and regulated thiol metabolism in rice under As stress which helped in restriction of As translocation (Das et al., 2021). Supplementation of Se decreases the uptake and translocation of As *via* altering root morphology, which is stimulated by ethylene (Feng et al., 2021).

Mustard plant is the third important oilseed crop in the world and second most important edible oilseed in India after groundnut contributing 28.6% to the total production of oilseeds and 27.8% in the India's oilseed economy (Shekhawat et al., 2012). Despite a large area under cultivation of mustard, its productivity is declining because of various abiotic stress factors which is a major challenge for modern agriculture (Gill et al., 2012). Mustard is an ideal system for assessing metal/metalloid toxicity and also known as a good accumulator of heavy metals (Pandey et al., 2016). This study was undertaken to integrate information on the effects of ethylene and Se in augmenting the photosynthetic activity of mustard under As stress in mustard. The potential for exogenously-sourced ethylene (as ethephon; 2-chloroethylphosphonic acid) to protect photosynthetic performance and plant dry mass was tested, and the involvement of ABA and the antioxidant system was investigated. It was postulated that ethylene induces Se availability for higher production of antioxidants, together with its own regulation of ABA synthesis and the activity and expression of antioxidant enzymes. The ethylene action inhibitor norbornadiene (NBD) was also utilized to substantiate the action of ethylene in amelioration of As stress.

## Materials and Methods

### Experimentation

Mustard (*Brassica juncea* L. Czern & Coss. var. Varuna) seeds obtained from the National Seeds Corporation, New Delhi, India were treated with 0.01% HgCl_2_, washed with double-distilled water and sown in clay pots of 23-cm diameter filled with peat and compost, 4:1 (v/v) together with sand, 3:1 (v/v). The cultivar Varuna was selected for the study as this has shown high photosynthetic potential of metal tolerance in our earlier study (Gill et al., 2011; Khan et al., 2016). The experiment was conducted in the Department of Botany, Aligarh Muslim University, Aligarh, India under natural day/night conditions, a temperature of 22°C/14°C (±3°C), photosynthetically active radiation (PAR) of 660 μ mol m^−2^ s^−1^, and a relative humidity of 62 ± 5%.

The influence of 200 μl L^−1^ ethephon (2-chloroethylphosphonic acid) and 2-mg Se kg^−1^ soil was tested for their effects on the amelioration of 24-mg As kg^−1^ soil induced adverse effects on photosynthesis and plant growth. Soil was thoroughly mixed with 2-mg Se kg^−1^ soil (as sodium selenite) and left for 15 days, while 24-mg As kg^−1^ soil (sodium arsenate) was supplied at the time of sowing. Ethephon at 200 μl L^−1^ (25 ml) was applied to plant foliage at 20 days after sowing (DAS). The ethylene action inhibitor NBD at 100 μM was applied on plants at 20 DAS to substantiate the role of ethylene in Se-mediated As stress mitigation. The concentration of ethephon, Se, As, and NBD was selected based on the prior studies (Khan et al., 2015, 2021b; Fatma et al., 2021; Sehar et al., 2021). On alternate days, plants were given the Hoagland nutrient solution (300 ml) to provide nutrients. The observations on different parameters were made at 30 DAS. The complete randomized block design (CRBD) was used for the treatment arrangement, and four replicates (*n* = 4) were maintained for each treatment.

### Determination of As Content

Leaf and root samples were oven-dried for 48 h at 80°C and ground into fine powder, which was then digested with concentrated HNO_3_/HClO_4_ (3:1, v/v). The As content was determined in samples by Atomic Absorption Spectrophotometer (GBC, 932 plus, GBC Scientific Instruments, Braeside, Australia). The procedure has been described earlier (Asgher et al., 2021).

### Translocation Factor

The translocation factor (TF) was calculated as the ratio of the metal concentration in leaves of the plant to that in roots using the following formula as reported earlier in Asgher et al. (2022).


$$\begin{array}{l} \text{TF }=\;\frac{\text{As concentration in leaf}}{\text{As concentration in root}} \end{array}$$

### Content of H_2_O_2_ and Thiobarbituric Acid Reactive Substances

The methods of Dhindsa et al. (1981) and Okuda et al. (1991) were used for estimating H_2_O_2_ and thiobarbituric acid reactive substances (TBARS) contents, respectively. The reaction mixture consisted of 1.5 ml eluate, 400-μl of 12.5 mM 3-dimethyl aminobenzoic acid (DMAB) in 0.375 M phosphate buffer (pH 6.5), 80 μl of 3-methyl-2-benzothiazoline hydrazone and 20 μl of peroxidase (0.25 unit). Peroxidase was added to start the reaction and increase in absorbance was recorded at 590 nm.

The homogenization medium for 500-mg fresh leaves contained 0.25% 2-thiobarbituric acid in 10% trichloroacetic acid. The mixture was heated at 95°C for 30 min and then was rapidly cooled on ice bath, followed by centrifugation at 10,000 g for 10 min. In 1.0-ml aliquot, 4.0 ml of 20% trichloroacetic acid containing 5% thiobarbituric acid was added. Finally, the color intensity was recorded at 532 nm.

The details of the methods are reported in Asgher et al. (2021) and included in Supplementary Material S1.

### Antioxidant Enzyme Activity

The methods adopted by Sehar et al. (2020) were used for estimation of the activity of superoxide dismutase (SOD), ascorbate peroxidase (APX), and glutathione reductase (GR). The extraction buffer containing 0.05% (v/v) Triton X-100 and 1% (w/v) PVP in potassium-phosphate buffer (100 mM, pH 7.0) was used to homogenize 200-mg leaves in chilled mortar and pestle. The centrifugation was done at 15,000 g for 20 min at 4°C. The supernatant thus collected was used for the assay of SOD (EC; 1.15.1.1) and GR (EC; 1.6.4.2) enzymes. The addition of 2.0-mM ascorbate with the extraction buffer was made for the assay of APX (EC; 1.11.1.11).

Supplementary Material S1 provides the details of the methods.

### Determination of GSH

The modified method of Anderson (1985) was used for determining GSH content. The homogenization of fresh leaf tissue (0.5 g) was done in 2.0 ml of 5% sulphosalicylic acid at 4°C at 10,000 g for 10 min. The supernatant (0.5 ml) was taken and 0.6 ml of phosphate buffer (100 mM, pH7.0) and 40 ml of 5/5/-dithiobis-2-nitro benzoic acid (DTNB) were added. After 2 min, the absorbance was recorded at 412 nm.

The details of the methods are included in Supplementary Material S1.

### Activity of 1-Aminocyclopropane-Carboxylic Acid Synthase and Ethylene Evolution

The methods of Avni et al. (1994) and Woeste et al. (1999) were used for estimating the activity of ACS (EC, 4.4.1.14). The homogenized preparation from leaf tissue (5.0 g) grounded in 100 mM HEPES buffer (pH 8.0) containing 4 mM DTT, 2.5 mM pyridoxal phosphate, and 25% PVP was centrifuged at 12,000 g for 15 min. In a 30 ml tube 1.0 ml of the supernatant and 0.1 ml of 5 mM S-adenosyl methionine (AdoMet) were added, and incubated for 2 h at 22°C. In the reaction, the ACC formed was determined by its conversion to ethylene by the addition of 0.1 ml of 20 mM HgCl_2_ followed by the addition of 0.1 ml of a 1:1 mixture of saturated NaOH/NaCl and placed on ice for 10 min. In the control set, AdoMet was not added. Ethylene evolution was determined on a gas chromatograph (Nucon 5700, New Delhi, India) able with a 1.8 m porapack N (80–100 mesh) column, a flame ionization detector and data station. Nitrogen was used as the carrier gas. The flow rates of hydrogen, nitrogen, and oxygen were 30, 30, and 300 ml min^−1^, respectively, the detector was set at 150°C. Ethylene was detected based on retention time and estimated by comparison with peaks from standard ethylene concentration.

The details of the procedure are reported by Fatma et al. (2021) and given in Supplementary Material S1.

### Abscisic Acid Determination

The determination of ABA was made by the method of Hung and Kao (2003) with slight modifications. ABA was determined with an ABA immunoassay detection kit (PGR-1; Sigma–Aldrich, St. Louis, MO, USA) as per the user manual. The ABA content was estimated from a calibration curve plotted by using standard ABA and values were recorded at 405 nm. The procedure is given in Fatma et al. (2021), and the details are included in Supplementary Material S1.

### Histochemical Staining

The procedure of Rather et al. (2020) was used to perform histochemical staining for the identification of superoxide ion and H_2_O_2_ ion accumulation. The nitroblue tetrazolium (NBT) and 3,3′-diaminobenzidine (DAB) were used for the assay of superoxide ion (O${}_{2}^{-}$) and H_2_O_2_ accumulation in the samples. The samples from each treatment were kept in NBT solution prepared by dissolving 0.1 g NBT in 50 ml of 50 mM sodium phosphate buffer (pH 7.5) in an amber-colored bottle and were kept overnight. The stained samples were dipped in absolute ethanol and boiled in a water bath for 10 min for discoloration to get the staining clear.

The DAB (50 mg) dissolved in 50 ml double-distilled water in an amber colored bottle with a pH in 3.8 was used for staining. The samples from each treatment were kept in DAB staining solution and incubated it for 8 h. The stained samples were dipped in absolute ethanol and boiled in a water bath for 10 min for discoloration to visualize the staining clearly. Supplementary Material S1 contains the detailed procedure.

### Determination of Gas Exchange Parameters, Rubisco Activity, Chlorophyll Fluorescence and Growth

Photosynthetic rate, stomatal conductance, and intercellular CO_2_ concentration were measured in fully exposed leaves using an infrared gas analyzer (CID-340, photosynthesis system, Bioscience, Camas, WA, USA). The measurements were taken between 11 a.m. and 12 p.m at a light saturating intensity (PAR) of 680 μmol CO_2_ m^−2^ s^−1^, temperature of 22°C, and relative humidity of approximately 70%. Chlorophyll content was determined with a SPAD chlorophyll meter (502 DL PLUS, Spectrum Technologies, Plainfield, IL, USA). The activity of Rubisco was determined by monitoring the NADH oxidation at 30°C at 340 nm when 3-phosphoglycerate is converted into glycerol-3-phosphate after the addition of enzyme extract to the reaction mixture. Rubisco was assayed as described earlier by Sehar et al. (2020). The details are given in Supplementary Material S1. Prior to assays, plants were cleaned and dry weights determined after drying at 80°C. The leaf area was measured using a leaf area meter (LA211, Systronics, New Delhi, India).

A Junior-PAM chlorophyll fluorometer (Heinz Walz, GmbH, Effeltrich, Germany) was used to assess chlorophyll fluorescence parameters, actual PSII efficiency, maximal quantum yield efficiency of PSII, intrinsic PSII efficiency, photochemical quenching (qP), non-photochemical quenching (NPQ), and electron transport rate. Details of the procedure were described by Sehar et al. (2020).

### Quantitative Real-Time Reverse Transcription–Polymerase Chain Reaction Analysis

Total RNA was extracted from fresh leaves (100 mg) of plants using Trizol reagent (Invitrogen, Carlsbad, CA, USA) according to the instructions given in the kit. Impurities in the extracted RNA were eliminated using an RNase-free DNase kit (Qiagen) and yields measured using a microplate reader (Biotek Instruments, Inc., USA). On a formaldehyde gel electrophoresis RNA integrity was determined by separating an equal volume of each sample (2 g). For the synthesis of first-strand complementary DNA, a Verso cDNA synthesis kit (Thermo Scientific) was used on DNA-free total RNA (1 g) according to the manufacturer's protocol. To assess gene expression, specific primers were designed as given in Supplementary Table S1. Quantitative real-time–polymerase chain reaction (qRT–PCR) was performed using Maxima SYBR Green/ROX qPCR Master Mix (2X) (Thermo Scientific) on a Light Cycler (Model 480, Roche, Germany). Biological and technical replicates were included in qRT–PCR reactions. In analyses, the target gene expression was normalized to actin expression and assessed using the 2^−ΔΔCt^ calculation method (Livak and Schmittgen, 2001).

### Statistical Analysis

Analysis of variance (ANOVA) was performed using the software SPSS 17.0 for Windows (SPSS Inc., Chicago, III., USA) to determine significant differences in the data. The data were presented as treatment mean ± SE (*n* = 4). The differences in the treatments were declared significant on the basis of the least significant difference (LSD) at *p* < 0.05. Bars with the same letter did not differ significantly by LSD test at *p* < 0.05.

## Results

### Effect of Ethephon and Selenium on As Content in Roots and Leaves

**Figure 1:**
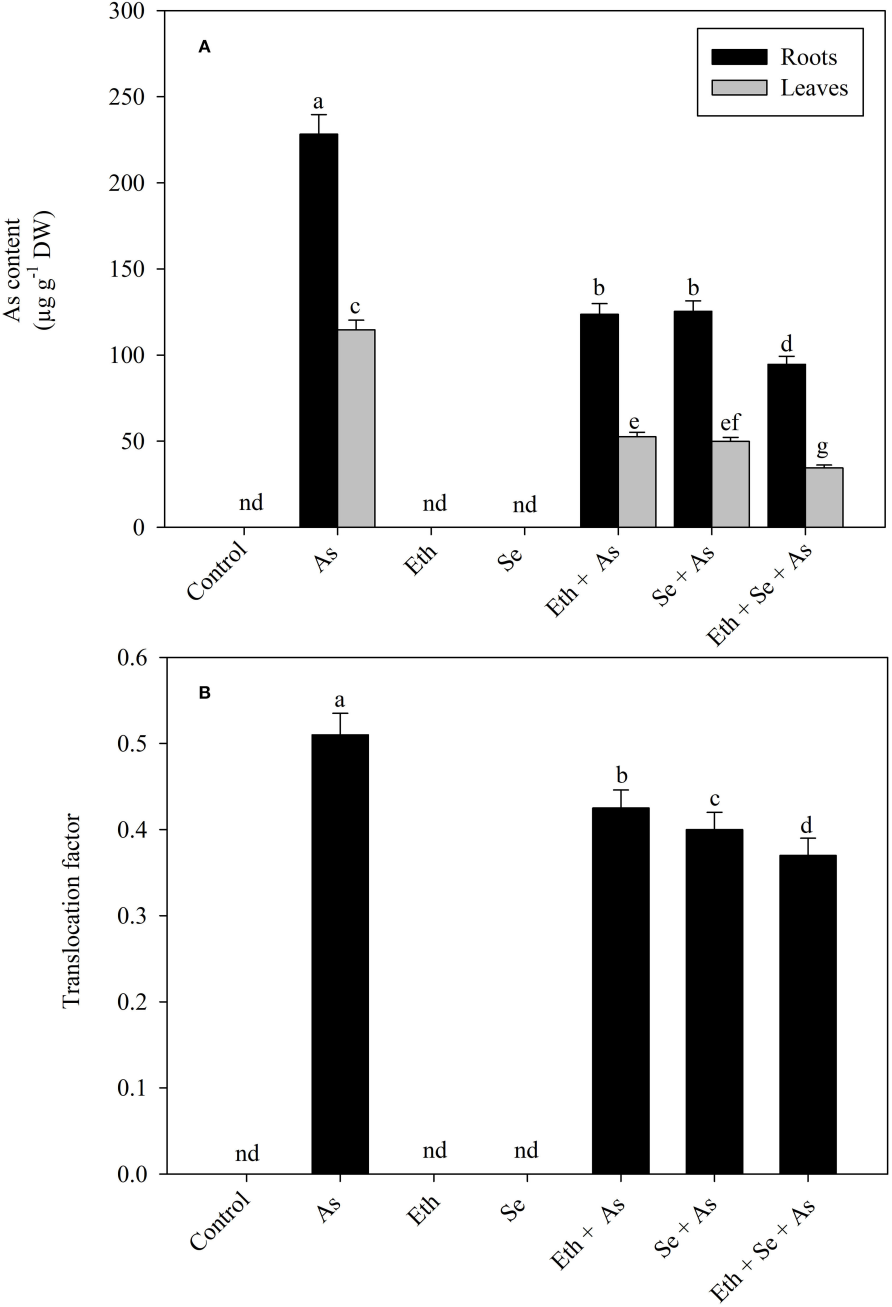
Content of As **(A)** in roots and leaves and TF **(B)** of mustard (*B. juncea* L.) grown with 200 μl L^−1^ Eth and/or 2-mg Se kg^−1^ soil in the presence or absence of 24-mg As kg^−1^ soil at 30 DAS. Data are presented as treatment mean ± SE (*n* = 4). Data followed by the same letter are not significantly different by LSD test at *p* < 0.05. As, arsenic; Eth, ethephon; Se, selenium.

Plants grown with As manifested significantly increased As accumulation in both roots and leaves and also exhibited significantly higher TF as compared to control plants. Individual application of ethephon and Se helped to reduce As content in roots and leaves, but maximal significant reduction was observed when treating with ethephon and Se together. Similar trend was observed with the TF. Specifically, relative to solely As-treated plants, application of either ethephon or Se reduced As content significantly in roots by 45.8% and in leaves by 54.1% and TF by 119.7%, but plants receiving ethephon plus Se showed As contents in roots and leaves maximally reduced by 58.5 and 70.3%, and TF by 127.5%, respectively (Figures 1A,B).

### Effect of Ethephon and Selenium on Oxidative Stress Under As Stress

To examine the effect of ethephon and Se on As-induced oxidative damage, oxidative stress parameters such as H_2_O_2_ and TBARS content were assessed. Plants grown with As showed significantly increased content of H_2_O_2_ by 88.3% and TBARS by 156.0% compared to control plants. Individual application of ethephon and Se each attenuated oxidative stress, decreasing H_2_O_2_ and TBARS similarly—ethephon by about 46.1% and Se by 31.1% in comparison to control plants. In the presence of As, both ethephon and Se equally decreased H_2_O_2_ and TBARS content. However, plants receiving ethephon and Se in the presence of As exhibited maximal significant decrease in H_2_O_2_ and TBARS contents, both being about 74.6% less than in As-treated plants (Table 1).

**Table 1:** The H_2_O_2_ content (nmol g^−1^ FW), TBARS content (nmol g^−1^ FW), SOD activity (U mg^−1^ protein min^−1^), APX activity (U mg^−1^ protein min^−1^), and GR activity (U mg^−1^ protein min^−1^) of mustard plant (*B. juncea* L.) grown with 200 μl L^−1^ Eth and/or 2 mg Se kg^−1^ soil in the presence or absence of 24 mg As kg^−1^ soil at 30 DAS.

**Treatments**	**H_**2**_O_**2**_ content**	**TBARS content**	**SOD activity**	**APX activity**	**GR activity**
Control	18.8 ± 0.94^b^	05.0 ± 0.26^b^	6.67 ± 0.32^g^	2.13 ± 0.16^g^	0.18 ± 0.009^g^
As	35.4 ± 1.68^a^	12.8 ± 0.64^a^	11.5 ± 0.56^f^	3.78 ± 0.21^de^	0.26 ± 0.013^def^
Eth	10.6 ± 0.52^e^	3.28 ± 0.16^e^	13.4 ± 0.66^d^	4.2 ± 0.25^d^	0.31 ± 0.015^d^
Se	11.4 ± 0.55^d^	3.46 ± 0.17^e^	12.7 ± 0.24^de^	3.35 ± 0.16^f^	0.28 ± 0.014^de^
Eth + As	16.6 ± 0.81^bc^	03.9 ± 0.18^cd^	18.2 ± 0.89^b^	7.32 ± 0.38^bc^	0.34 ± 0.017^bc^
Se + As	17.5 ± 0.86^b^	04.3 ± 0.21^c^	17.4 +0.85^bc^	7.9 ± 0.4^b^	0.38 ± 0.019^b^
Eth + Se + As	09.3 ± 0.45^f^	2.94 ± 0.14^f^	22.4 ± 1.07^a^	9.8 ± 0.57^a^	0.44 ± 0.022^a^

*Data are presented as treatment mean ± SE (n = 4). Data followed by same letter are not significantly different by LSD test at p < 0.05. As, arsenic; APX, ascorbate peroxidase; GR, glutathione reductase; H_2_O_2_, hydrogen peroxide; Eth, ethephon; Se, selenium; SOD, superoxide dismutase; TBARS, thiobarbituric acid reactive substances*.

### Effect of Ethephon and Selenium on Antioxidant Enzymes Under As Stress

The antioxidant capacity of plants grown with As after treatment with ethephon and Se treatment was evaluated by determining activity of SOD, APX, and GR. Exposure to As significantly stimulated SOD, APX, and GR activity, with respective values of 72.4, 77.4, and 44.4% over those of control plants. Plants receiving ethephon or Se in the presence of As further conspicuously upregulated activity of these antioxidant enzymes in comparison to control or As treated plants, while maximum significant increase in antioxidative enzymes occurred with ethephon application together with Se (Table 1).

### Effect of Ethephon and Selenium on GSH Content Under As Stress

**Figure 2:**
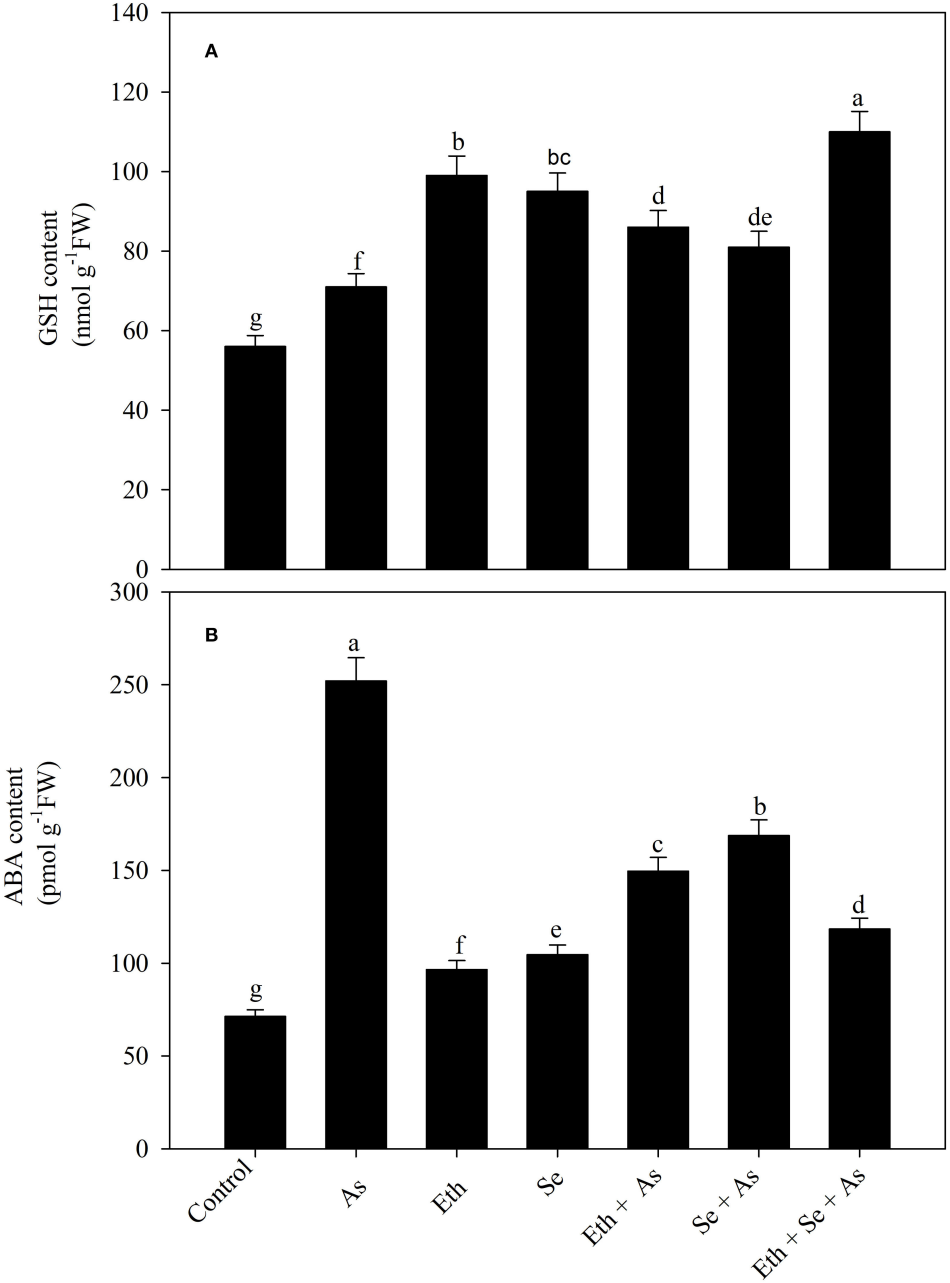
Foliar content of GSH **(A)** and ABA **(B)** in mustard (*B. juncea* L.) grown with 200 μl L^−1^ Eth and/or 2-mg Se kg^−1^ soil in the presence or absence of 24-mg As kg^−1^ soil at 30 DAS. Data are presented as treatment mean ± SE (*n* = 4). Data followed by the same letter are not significantly different by LSD test at *p* < 0.05. ABA, abscisic acid; As, arsenic; Eth, ethephon; GSH, reduced glutathione; Se, selenium.

Arsenic stress significantly increased GSH content as compared to control plants. Individual treatment with ethephon/Se significantly increased likewise GSH content, in both cases by 78.1% in comparison to control plants. Similarly, in the presence of As, both ethephon or Se caused significantly equal increase in GSH content relative to control plants, but maximum significant increase was observed with combined ethephon and Se content which was 96.4% compared to control plants (Figure 2A).

### Effect of Ethephon and Selenium on ABA Content Under As Stress

Plants exposed to As exhibited significant increase in ABA content of 253.4% in comparison with controls. Those treated with ethephon or Se exhibited approximately similar trends whether in the presence or absence of As, with ABA content being decreased relative to As-exposed plants. Notably, plants receiving both ethephon and Se in the presence of As exhibited significantly decreased ABA (53.1%) in comparison to those exposed to As alone (Figure 2B).

### Effect of Ethephon and Selenium on 1-Aminocyclopropane Carboxylic Acid Synthase Activity and Ethylene Evolution Under As Stress

**Figure 3:**
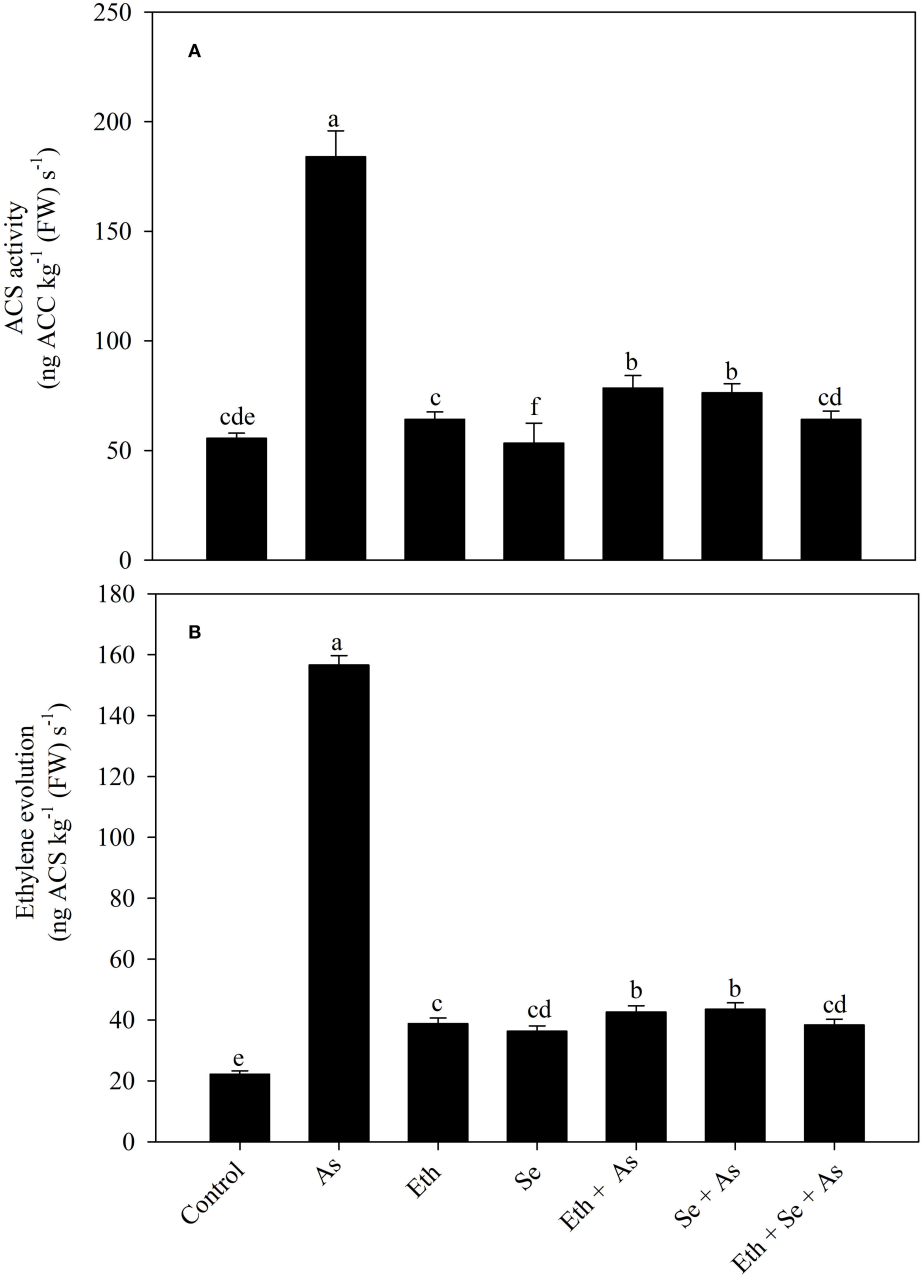
Activity of ACS **(A)** and ethylene evolution **(B)** in mustard (*B. juncea* L.) leaves grown with 200 μl L^−1^ Eth and/or 2-mg Se kg^−1^ soil in the presence or absence of 24-mg As kg^−1^ soil at 30 DAS. Data are presented as treatment mean ± SE (*n* = 4). Data followed by the same letter are not significantly different by LSD test at *p* < 0.05. ACS, 1-aminocyclopropane carboxylic acid; As, arsenic; Eth, ethephon; Se, selenium.

Plants grown in the presence of As exhibited greater 1-aminocyclopropane carboxylic acid synthase (ACS) activity and ethylene evolution, respectively, increased by 3.3 times (230.9%) and 7.1 times (605.1%) relative to control plants. Individual application of ethylene and Se each increased ACS activity and ethylene evolution as compared to control plants, but decreased values relative to As-treated plants. In the presence of As, supplementation with ethephon or Se decreased the effect of As and likewise equally decreased ACS activity and ethylene evolution relative to As-stressed plants, by 58.1 and 70.3%, respectively. Supplementation of As-stressed plants with both ethephon and Se resulted in maximum significant decreases in ACS activity and ethylene evolution, by 65.2 and 75.8% relative to As-stressed plants (Figure 3).

### Effect of Ethephon and Selenium on Histochemical Staining Under As Stress

**Figure 4:**
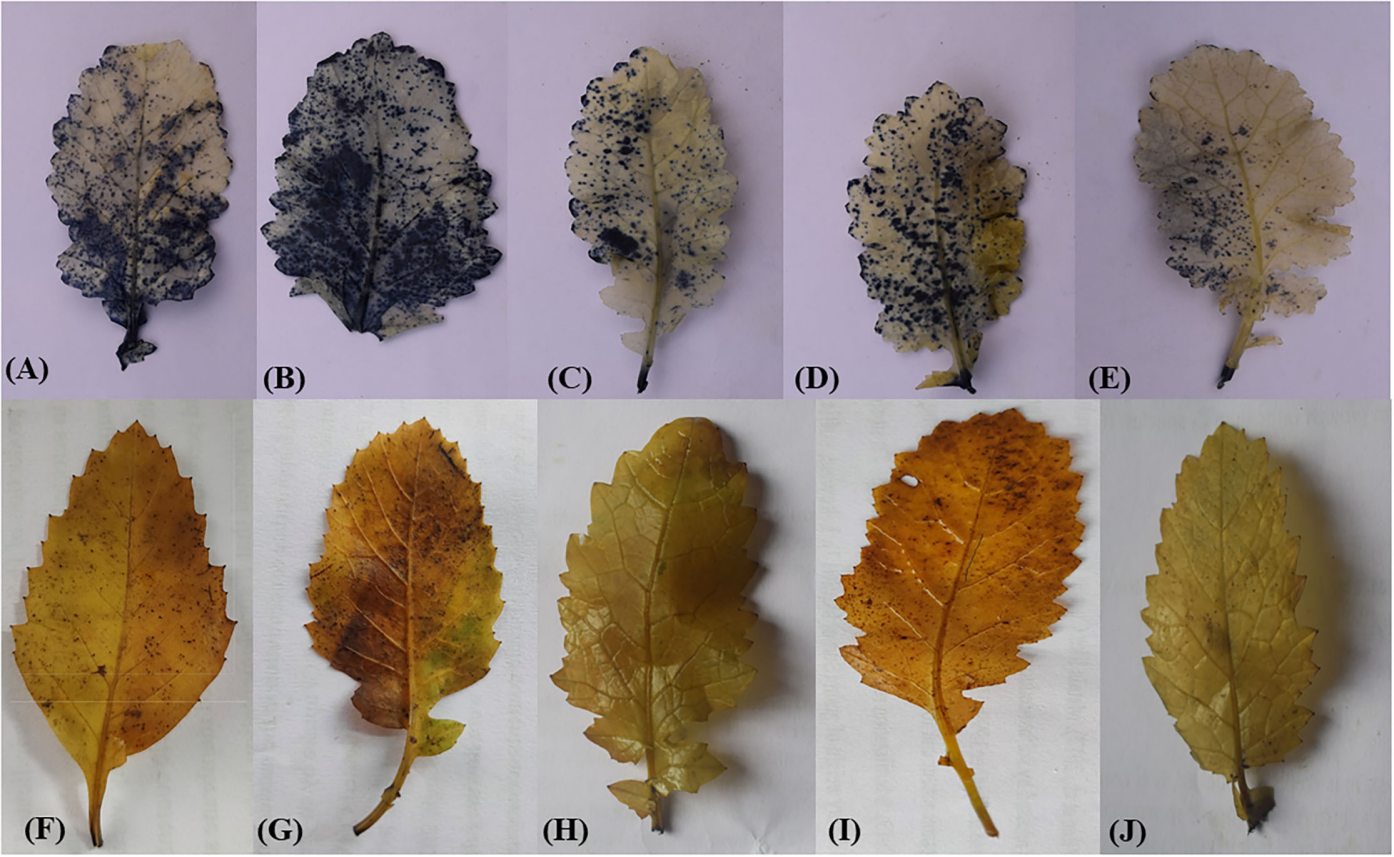
*In situ* determination of generation of O${}_{2}^{-}$ by NBT **(A**–**E)**, H_2_O_2_ by DAB **(F**–**J)** staining, respectively, of mustard (*B. juncea* L.) at 30DAS. Plants were treated with control **(A)** and **(F)**; As **(B)** and **(G)**; Eth + As **(C)** and **(H)**; Eth + Se **(D)** and **(I)**; Eth + Se + As **(E)** and **(J)**. A single representative of each treatment is shown. As, arsenic; DAB, diaminobenzidine; Eth, ethephon; H_2_O_2_, hydrogen peroxide; NBT, nitroblue tetrazolium; Se, selenium.

Accumulation of ROS in treated plants was monitored histochemically using NBT and DAB dyes (Figures 4A–J). An NBT staining detected the accumulation of O${}_{2}^{-}$ in leaves, which was measured in terms of the dark–blue stained area. Leaves from As-treated plants possessed more evident dark–blue spots relative to leaves from control plants, which clearly indicated greater O${}_{2}^{-}$ accumulation under As stress (Figure 4B). Remarkably, plants given the combined ethephon and Se treatment showed fewer dark–blue spots relative to those given either treatment alone, which supports an ameliorative role of ethephon and Se in As-induced oxidative stress (Figure 4E). DAB staining detected the accumulation of H_2_O_2_ as measured through the formation of dark-brown formazan (Figures 4F,J). Leaves from As-stressed plants showed deep brown spots clearly indicating H_2_O_2_ deposits (Figure 4G). Plants treated with either ethephon or Se showed significantly less accumulation of H_2_O_2_ in comparison to As-stressed plants; however, no brown-colored spots were evident in the leaves of As-exposed plants given the combined treatment (Figure 4J).

### Effect of Ethephon and Selenium on Chlorophyll Fluorescence Parameters Under As Stress

In plants receiving As, actual PSII efficiency was significantly decreased by 33.3%, maximal quantum yield efficiency of PSII by 15.7%, intrinsic PSII efficiency by 8.1%, qP by 27.0%, and electron transport rate by 33.3%; however, NPQ increased by 14.5% relative to control plants. Individual application of ethephon and Se improved the above parameters when compared to both control and As-treated plants, and reduced the effect of As stress. However, significant maximal amelioration of As stress was found with combined application of ethephon and Se; the joint treatment significantly allayed As-induced toxicity and resulted in increased actual PSII efficiency, maximal quantum yield efficiency of PSII, intrinsic PSII efficiency, qP and electron transport rate, but decreased NPQ relative to control plants (Table 2).

**Table 2:** Actual PSII efficiency, maximal quantum yield of PSII efficiency, intrinsic PSII efficiency, qP, NPQ, and electron transport rate of mustard (*B. juncea* L.) grown with 200 μl L^−1^ Eth and/or 2-mg Se kg^−1^ soil in the presence or absence of 24-mg As kg^−1^ soil at 30 DAS.

**Treatments**	**Actual PSII efficiency**	**Maximal quantum yield efficiency of PSII**	**Intrinsic PSII efficiency**	**Qp**	**NPQ**	**Electron transport rate**
Control	0.78 ± 0.03^cd^	0.76 ± 0.04^cd^	0.73± 0.04^cd^	0.77 ± 0.04^d^	0.62 ± 0.03^c^	199.6 ± 9.2^c^
As	0.52 ± 0.02^e^	0.64 ± 0.03^e^	0.68 ± 0.03^e^	0.54 ± 0.03^f^	0.71 ± 0.04^a^	149.7 ± 7.8^e^
Eth	0.81 ± 0.03^ab^	0.81 ± 0.04^b^	0.78 ± 0.04^b^	0.83 ± 0.04^b^	0.54 ± 0.03^de^	207.7 ± 10.7^ab^
Se	0.80 ± 0.03^bc^	0.79 ± 0.04^bc^	0.77 ± 0.04^bc^	0.82 ± 0.04^bc^	0.59 ± 0.03^d^	204.5 ± 10.5^b^
Eth + As	0.79 ± 0.03^c^	0.77 ± 0.08^c^	0.74 ±0.03^c^	0.80 ± 0.04^c^	0.65 ± 0.03^bc^	196.5 ± 9.6^c^
Se + As	0.77 ± 0.03^d^	0.74 ± 0.38^d^	0.70 +0.03^d^	0.75 ± 0.03^e^	0.67 ± 0.035^b^	193.5 ± 9.5^cd^
Eth + Se + As	0.82 ± 0.03^a^	0.83 ± 0.05^a^	0.80 ± 0.04^a^	0.86 ± 0.04^a^	0.51 ± 0.03^e^	217.6 ± 10.1^a^

*Data are presented as treatment mean ± SE (n = 4). Data followed by same letter are not significantly different by LSD test at p < 0.05. As, arsenic; Eth, ethephon; NPQ, non-photochemical quenching; qP, photochemical quenching; Se, selenium*.

### Effect of Ethephon and Selenium on Photosynthetic and Growth Attributes Under As Stress

Plants exposed to As stress exhibited significant declines in net photosynthesis (by 43.5%), intercellular CO_2_ concentration (by 23.2%), stomatal conductance (by 31.8%), chlorophyll content (by 32.3%), and Rubisco activity (by 39.1%) compared to control plants. Under individual ethephon and Se supplementation, all photosynthetic indices increased equally. In the presence of As, ethephon and Se application were likewise equally effective in significantly stimulating photosynthetic attributes and reversing the effects of As, but the highest values were observed in plants receiving combined application of ethephon and Se, which more prominently alleviated the negative impacts of As and significantly enhanced net photosynthesis, intercellular CO_2_ concentration, stomatal conductance, chlorophyll content, and Rubisco activity (Table 3).

**Table 3:** Chlorophyll content (SPAD value), Rubisco activity (μmol CO_2_ mg^−1^ protein min^−1^), stomatal conductance (mmol CO_2_ m^−2^ s^−1^), intercellular CO_2_ concentration (μmol CO_2_ mol^−1^), net photosynthesis (μmol CO_2_ m^−2^ s^−1^), leaf area (cm^2^ plant^−1^), and plant dry mass (g plant^−1^) of mustard (*B. juncea* L.) grown with 200 μl L^−1^ Eth and/or 2-mg Se kg^−1^ soil in the presence or absence of 24-mg As kg^−1^ soil at 30 DAS.

**Treatments**	**Chlorophyll content**	**Rubisco activity**	**Stomatal conductance**	**Intercellular CO_**2**_ concentration**	**Net photosynthesis**	**Leaf area**	**Plant dry mass**
Control	32.8 ± 1.61^de^	0.74 ± 0.039^ef^	367 ± 11.4^d^	258 ± 10.11^e^	13.8 ± 0.66^ef^	108.4 ± 5.4^e^	2.20 ± 0.11^ef^
As	22.2 ± 1.09^f^	0.45 ± 0.022^g^	282 ± 09.4^f^	176 ± 07.01^f^	7.80 ± 0.35^g^	058.4 ± 2.8^f^	0.89 ± 0.01^g^
Eth	43.5 ± 2.12^ab^	1.04 ± 0.053^b^	362 ± 14.8^d^	360 ± 14.6^b^	22.4 ± 1.08^ab^	154.5 ± 7.6^b^	2.93 ± 0.15^b^
Se	41.6 ± 2.07^bc^	0.96 ± 0.048^bc^	358 ± 14.2^de^	354 ± 14.2^b^	21.6 ± 1.04^bc^	151.3 ± 7.4^b^	2.78 ± 0.14^bc^
Eth + As	35.4 ± 1.69^d^	0.88 ± 0.043^cd^	445 ± 17.2^b^	324 ± 13.8^c^	17.8 ± 0.84^d^	138.5 ± 6.8^bc^	2.45 ± 0.12^d^
Se + As	34.8 ± 1.64^d^	0.81 ± 0.04^cde^	428 ± 16.3^bc^	304 ± 12.6^cd^	16.6 ± 0.79^de^	132.4 ± 6.5^cd^	2.38 ± 0.12^de^
Eth + Se + As	47.5 ± 2.31^a^	1.28 ±0.066^a^	542 ± 21.6^a^	389 ± 15.1^a^	24.2 ± 1.18^a^	184.6 ± 9.1^a^	3.48 ± 0.18^a^

*Data are presented as treatment mean ± SE (n = 4). Data followed by same letter are not significantly different by LSD test at p < 0.05. As, arsenic; Eth, ethephon; Rubisco, ribulose, 1,5- bisphosphate carboxylase/oxygenase; Se, selenium*.

As-stressed plants exhibited significant reductions in the leaf area and plant dry mass, of 46.1 and 59.5%, respectively, when compared to controls. In contrast, those treated with ethephon or Se alone showed increased dry mass (by 33.1 and 26.4%, respectively) and leaf area (by 42.5 and 39.6%, respectively) relative to controls. In addition, the negative effects of As on plant growth were greatly improved with individual application of ethephon or Se, though the combined application of ethephon and Se proved more effective, increasing plant dry mass and leaf area by 3.9 and 3.2 times, respectively, relative to plants exposed to As alone (Table 3).

### Effects of NBD on ABA Content, Oxidative Stress, and the Activity and Expression of Antioxidant Enzymes

Relative to controls and to As-stressed plants receiving combined ethephon/Se treatment, the application of NBD (an ethylene action inhibitor) to As-stressed plants either alone or in combination with ethephon plus Se resulted in increases of H_2_O_2_, TBARS, and ABA contents, with subsequent decreases in the activity and expression of antioxidant enzymes, stomatal conductance, net photosynthesis, and plant dry mass. This confirms that combined application of ethephon and Se alleviated As stress on photosynthesis by means involving ABA and antioxidant enzymes, which in turn led to an increase in photosynthesis *via* ethylene action on the stomata/guard cells while also lowering H_2_O_2_ and TBARS content.

**Figure 5:**
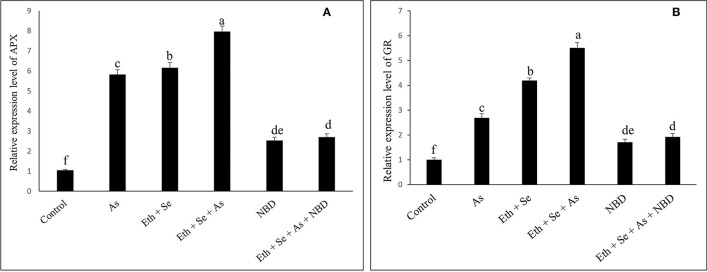
Relative expression level of genes encoding APX **(A)** and GR **(B)** enzymes in mustard (*B. juncea* L.) leaves grown with 200 μl L^−1^ Eth and/or 2-mg Se kg^−1^ soil in the presence or absence of 24-mg As kg^−1^ soil at 30 DAS. The treatment with 100 μM NBD was applied either alone or with combination of 200 μl L^−1^ Eth and 2-mg kg^−1^ soil Se with 24-mg As kg^−1^ soil. Results are represented relative to the respective control (control, 1). Data are represented as means ± SE (*n* = 4). Data followed by same letter are not significantly different by LSD test at *p* < 0.05. As, arsenic; Eth, ethephon; Se, selenium.

In the presence of As, application of ethephon plus Se significantly decreased ABA content by 77.5%, H_2_O_2_ content by 71.8%, and TBARS content by 76.2% relative to As treatment alone (Tables 4, 5). It also increased stomatal conductance by 50%, net photosynthesis by 77.7%, and plant dry mass by 58.5% relative to controls. However, maximal increases in activity of the antioxidant enzymes APX and GR were obtained with the combined application of ethephon and Se to plants under As stress. Application of NBD to As-stressed plants given the combined treatment increased ABA, H_2_O_2_, and TBARS content, resulting in decreased antioxidant enzyme activity, stomatal conductance, net photosynthesis, and plant dry mass. These findings highlight the role of ethylene in inhibiting ABA-mediated stomatal closure and photosynthesis (Tables 4, 5). Moreover, combined application of ethephon plus Se to plants under As stress significantly upregulated expression of the genes encoding APX and GR enzymes relative to both As treatment alone and the control (Figure 5). Treatment with NBD significantly reduced APX and GR expression whether alone or in combined application with ethephon and Se in plants under As stress (Figure 5).

**Table 4:** The ABA content (pmol g^−1^FW), stomatal conductance (mmol CO_2_ m^−2^ s^−1^), net photosynthesis (μmol CO_2_ m^−2^ s^−1^), and plant dry mass (g plant^−1^) of mustard plant (*B. juncea* L.) grown with 200 μl L^−1^ Eth and/or 2-mg Se kg^−1^ soil in the presence or absence of 24-mg As kg^−1^ soil at 30 DAS.

**Treatments**	**ABA**	**Stomatal conductance**	**Net photosynthesis**	**Plant dry mass**
Control	060.3 ± 5.0^cd^	360 ± 11.0^c^	13.5 ± 0.66^c^	2.17 ± 0.10^c^
As	240.0 ± 8.3^a^	278 ± 09.1^f^	7.76 ± 0.35^f^	0.85 ± 0.01^f^
Eth + Se	049.4 ± 4.1^f^	552 ± 22.0^a^	26.3 ± 1.19^a^	3.82 ± 0.20^a^
Eth + Se + As	054.0 ± 4.8^e^	540 ± 21.2^b^	24.0 ± 1.18^b^	3.44 ± 0.16^b^
NBD	061.9 ± 5.2^c^	328 ± 10.2^d^	12.4 ± 0.60^d^	2.07 ± 0.08^d^
Eth + Se + As + NBD	226.0 ± 8.1^b^	306 ± 9.40^e^	9.71 ± 0.58^e^	1.36 ± 0.06^e^

*The treatment with 100-μM NBD was applied either alone or with combination of 200 μl L^−1^ Eth and 2-mg kg^−1^ soil Se with 24-mg As kg^−1^ soil. Data are presented as treatment mean ± SE (n = 4). Data followed by same letter are not significantly different by LSD test at p < 0.05. ABA, Abscisic acid; As, arsenic; Eth, ethephon; FW, fresh weight; NBD, norbornadiene; Se, Selenium*.

**Table 5:** The H_2_O_2_ content (nmol g^−1^ FW), TBARS content (nmol g^−1^ FW), APX activity (U mg^−1^ protein min^−1^), and GR activity (U mg^−1^ protein min^−1^) of mustard plant (*B. juncea* L.) grown with 200 μl L^−1^ Eth and/or 2-mg Se kg^−1^ soil in the presence or absence of 24-mg As kg^−1^ soil at 30 DAS.

**Treatments**	**H_**2**_O_**2**_ content**	**TBARS content**	**APX activity**	**GR activity**
Control	17.6 ± 0.81^d^	05.3 ± 0.26^d^	2.11 ± 0.09^f^	0.16 ± 0.006^f^
As	34.4 ± 1.60^a^	12.2 ± 0.60^a^	4.08 ± 0.16^c^	0.25 ± 0.011^c^
Eth + Se	05.2 ± 0.58^f^	1.91 ± 0.08^f^	08.9 ± 0.51^b^	0.43 ± 0.022^b^
Eth + Se + As	09.7 ± 0.47^e^	2.90 ± 0.12^e^	10.1 ± 0.54^a^	0.47 ± 0.027^a^
NBD	22.9 ± 0.84^c^	09.8 ± 0.41^c^	2.80 ± 0.09^de^	0.19 ± 0.007^de^
Eth + Se + As + NBD	29.8 ± 1.55^b^	11.3 ± 0.56^b^	2.92 ± 0.11^d^	0.20 ± 0.008^d^

*The treatment with 100-μM NBD was applied either alone or with combination of 200 μl/L Eth and 2-mg kg^−1^ soil Se with 24-mg As kg^−1^ soil. Data are presented as treatment mean ± SE (n = 4). Data followed by same letter are not significantly different by LSD test at p < 0.05. APX, ascorbate peroxidase; As, arsenic; Eth, ethephon; FW, fresh weight; GR, glutathione reductase; H_2_O_2_, hydrogen peroxide; NBD, norbornadiene; Se, Selenium; TBARS, thiobarbituric acid reactive substances*.

## Discussion

Arsenic is a metalloid and group I carcinogen that is highly toxic to all life forms and poses a severe threat to plant growth and productivity (Tripathi et al., 2007; Srivastava et al., 2016; Asgher et al., 2021; Khan et al., 2021a,b). It enters the plant employing phosphate transporters, and the accumulation leads to the production of excess ROS and alterations in the activity of antioxidants (Tripathi et al., 2007; Srivastava et al., 2016; Asgher et al., 2021; Bali and Sidhu, 2021). Recent reports have shown that As stress damages the photosynthetic machinery and inhibits photosynthesis (Kaya et al., 2020; Asgher et al., 2021, 2022; Khan et al., 2021a,b). In this study, the negative influence of As stress was attributed to a more significant translocation of As in leaf and higher increase in its content in leaves than in roots, leading to more production of H_2_O_2_ and oxidative stress in leaves and thus negatively impacted photosynthetic and growth performance. This work demonstrated that As-inhibited photosynthetic performance could be maximally reduced by applying ethylene and Se that limited As uptake in leaves and enhanced efficiency of the antioxidant system.

This study found that plants exposed to As stress exhibited excessive ethylene production and reduced photosynthetic performance. This could be due to the formation of stress ethylene that caused photosynthetic repression. However, the application of ethephon and Se together maximally reduced stress ethylene and brought it to the optimal level that plant needed for positively influencing photosynthesis. The possible reason may be the optimal ethylene-induced maximum enhancement in antioxidant potential that reduced oxidative stress. Combined supplementation of ethylene (as ethephon) and Se has previously been shown to optimize ethylene formation, regulate the antioxidant defense system, and substantially regulate GSH production to alleviate the adverse effects of As toxicity on photosynthetic performance (Alam et al., 2019). In addition, Se reverses Cd-induced oxidative stress by regulating ethylene biosynthesis and higher GSH levels and improved photosynthetic efficiency by controlling stomatal conductance and diffusion of CO_2_ across intercellular spaces in *T. aestivum* (Khan et al., 2015). However, reports on the photosynthetic efficiency with combined supplementation of ethylene and Se under As stress are not available. The independent studies have shown that ethylene or Se could reduce oxidative stress by regulating ROS levels (Singh et al., 2018, 2021). In particular, the available Se helps produce more reduced-S compounds for detoxification of ROS. The role of Se in reducing the adverse effects of metalloids has previously been shown. Feng et al. (2021) have found that Se downregulates genes encoding proteins responsible for heavy metal uptake and translocation and decreases membrane transporters' activity. Selenium regulates ROS metabolism and the activity of antioxidant enzymes resulting in reduced damage to plants (Chauhan et al., 2019; Hasanuzzaman et al., 2022). Application of Se advanced Cd tolerance due to the increased GSH content in *B. napus* (Hasanuzzaman et al., 2012) and *T. aestivum* (Khan et al., 2015). Wang et al. (2021) have shown that Se nanoparticles (SeNPs) and selenite mitigated As toxicity in rice through inhibition of As translocation to shoot and reduced uptake in mungbean (Malik et al., 2012). Moreover, it has been shown that Se takes up the functions of S because of their similar chemical properties and benefits plants to reduce oxidative stress (Khan et al., 2015; Chauhan et al., 2019). Selenium and S are both involved in the overexpression of ATP-sulfurylase (Pilon-Smits et al., 1999). Thus, in this study, the interaction between Se and ethylene improved S-assimilation pathway that produced S-adenosyl methionine, the precursor of ethylene. Selenium and S compete for the same transporters in roots (Chauhan et al., 2019; Zhou et al., 2020). Chauhan et al. (2020) found that the supplementation of Se to As stressed plants increased the expression of sulfate transporters (SULTR3; 1 and SULTR3; 6) and upregulated the antioxidant enzymes to reduce As accumulation and oxidative stress. Further, ethylene raises the levels of sulfate transporters and S assimilatory genes (Wang et al., 2018). It has also been reported that ethylene and Se act interactively in defense responses to Cd stress by stimulating the antioxidant enzymes (Alves et al., 2019). Our findings support that ethylene was involved in Se uptake and limited As translocation to leaves together with the positive effects of Se to reduce oxidative stress through upregulation of expression of antioxidant enzymes. Also, ethephon induced GSH production to alleviate As toxicity, and ethylene supplemented with Se minimized As-induced oxidative stress through enhancement of antioxidant enzymes. Singh et al. (2021) found that ethylene impacted As stress tolerance by enhancing the expression of sulfate transporters group causing greater translocation of sulfate in shoot tissues resulting in higher GSH synthesis and As detoxification. The ethylene overproduction (*eto1-1*) and signaling (*ctr1-10*) mutant were more tolerant to As stress and ethylene was essential for As tolerance. In this study, the inhibition of ethylene action by NBD in the presence of ethephon plus Se and As increased oxidative stress and ABA content, and inhibited photosynthesis and plant dry mass. This signifies the involvement of ethylene in the presence of Se in reducing oxidative stress and protection of As-inhibited photosynthesis.

**Figure 6:**
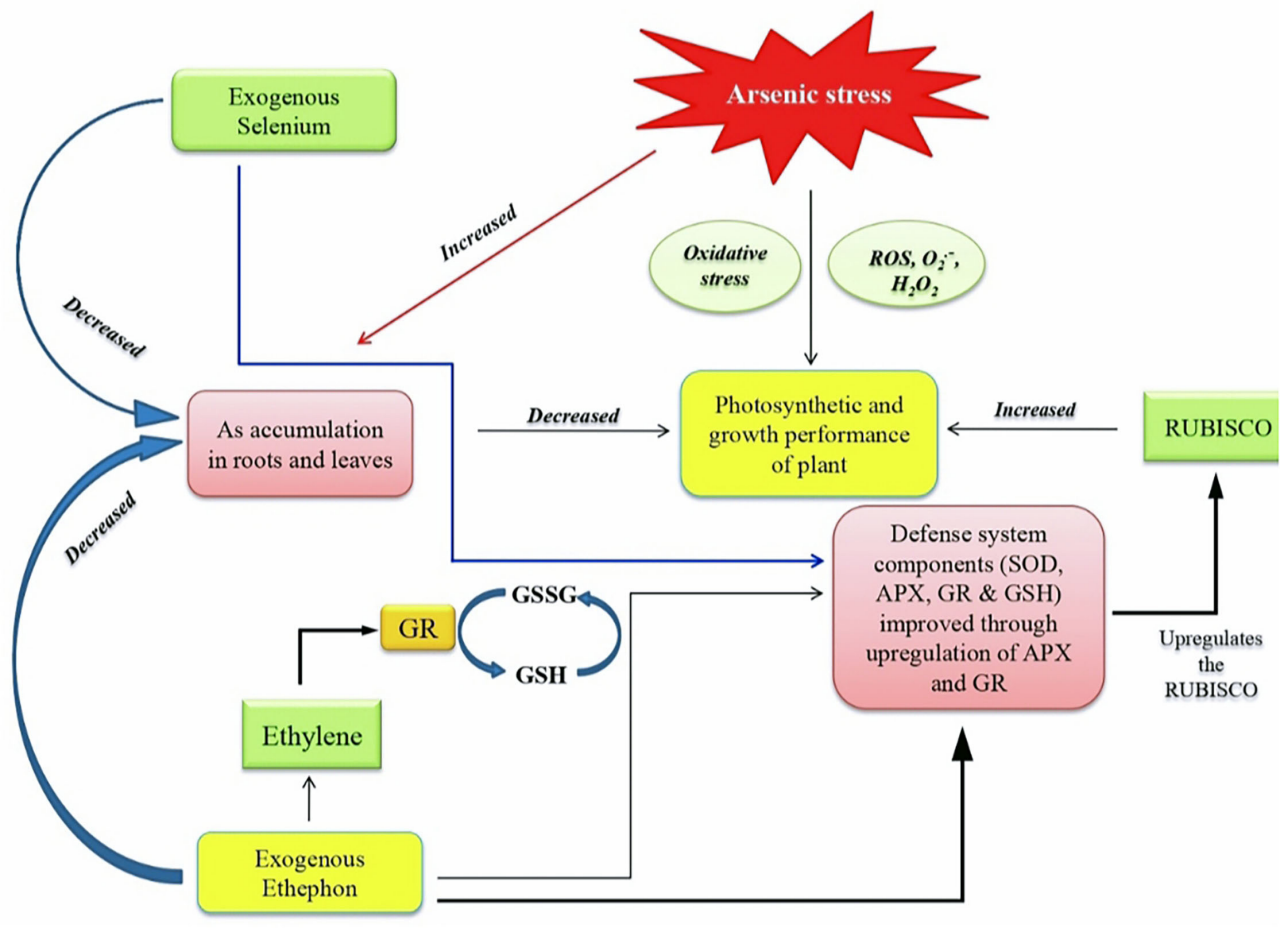
Model showing the impact of As on plants and its reversal employing ethephon (ethylene source) and selenium in plants. As, arsenic; ROS, reactive oxygen species; GSSG, oxidized glutathione, GSH, reduced glutathione; SOD, super oxide dismutase; APX, ascorbate peroxidase; GR, glutathione reductase; RUBISCO, ribulose-1,5-bisphosphate carboxylase/oxygenase.

Plants exposed to As stress exhibited increased ABA accumulation and stomatal closure. Both ethylene and ABA have antagonistic effects in stomatal regulation (Tanaka et al., 2013). In this study, the application of ethephon and Se resulted in reduced ABA accumulation and oxidative stress. Plants supplemented with ethylene and Se demonstrated improved plant photosynthetic performance due to the improvement in the efficiency of pigment system II, increased stomatal conductance and intercellular CO_2_ concentration. In this study, ethephon treatment resulted in ABA suppression to help stomatal opening that was partially closed in the presence of As stress for increased gas exchange and photosynthesis. It may be emphasized that ethylene regulated GSH levels and maintained cellular redox homeostasis for the regulation of stomatal movement. The report on the effect of ethephon and Se on photosynthetic machinery under As stress has not been shown in the literature. The increase in stomatal aperture by ethylene supplementation was due to its effect on ABA content in guard cells. Our research is backed up by the previous findings that showed exogenous application of ethylene and/or Se improves photosynthetic efficiency through increased stomatal conductance and the diffusion of CO_2_ across intercellular spaces in *B. juncea* (Iqbal et al., 2012; Khan et al., 2016) and *T. aestivum* (Khan et al., 2015). It has been shown that the expression of defense responsive genes was antagonistically controlled by ethylene signaling mutants and ABA synthesis pathway mutants (Beaudoin et al., 2000; Ghassemian et al., 2000; Yanagisawa et al., 2003). In this study, the combined application of ethephon and Se to plants under As stress produced an optimal ethylene level that suppressed ABA accumulation and regulated antioxidant system to counteract As toxicity. Similarly, Fatma et al. (2021) showed that the application of ethephon reduced ABA content and increased the activity of antioxidant enzymes in salt-stressed *B. juncea*. Another study showed that ABA and ethylene given together increased guard cells activity of antioxidant enzymes, resulting in lowered H_2_O_2_ and reduced stomatal closure (Beguerisse-Diaz et al., 2012). The roles of ethylene and ABA in upregulating stress–responsive genes and stimulating GSH production to minimize abiotic stress conditions were suggested by Kumar et al. (2016). Studies have shown the relationship between ethylene and Se in plants (Zhu et al., 2017; Hajiboland et al., 2019). Malheiros et al. (2019) found that Se partially inhibited the biosynthesis of ethylene in the roots of rice seedlings. This study further revealed that the combined application of ethephon and Se increased the activity and expression of genes encoding APX and GR and suppressed ABA synthesis, and consequently protected plants from As stress damage. These results were substantiated with the application of NBD, an ethylene action inhibitor. The NBD treatment either alone or combined with ethephon and Se under As stress increased accumulation of ABA, H_2_O_2_, and TBARS and decreased the activity and expression of antioxidant enzymes. The crosstalk between ethylene, ABA, and Se is not available under As stress. The summary of the study illustrated through a model (Figure 6) shows that both ethephon and Se reduced As uptake and increased antioxidant enzymes activity to scavenge ROS, reduced ABA content and increased Rubisco activity to enhance photosynthetic responses under As stress. Thus, supplementation of ethylene and Se in plants grown under As contaminated soil may be done to mitigate As toxicity and reverse photosynthetic inhibition *via* regulation of antioxidant system and hormone biosynthesis.

## Conclusions

In conclusion, As inhibited photosynthesis and plant growth by reducing PSII activity and influenced stomatal limitations. The combined application of ethephon with Se to plants under As stress maximally maintained photosynthesis by reducing oxidative stress through stimulation of the antioxidant defense system and GSH synthesis, and also lowering As and ABA levels in leaves. Inhibition of ethylene action by NBD revealed the role of ethylene in regulation of the antioxidant system as well as the structure and function of the photosynthetic apparatus. The treatment with NBD either alone or in conjunction with ethylene and Se under As stress was found to increase ABA content, suggesting that ethylene action is important in suppressing ABA content and ABA-mediated regulation of stomatal conductance. Thus, these findings suggested that ethylene and Se upregulated the activity and expression of genes encoding the antioxidant enzymes, APX and GR and decreased ABA accumulation in guard cells, that helped regulation of stomatal conductance, protection of the PSII assembly, and maintained photosynthetic performance under As stress. The outcome of this study suggests that supplementation of ethylene and Se may act as a potential tool to reduce As stress and the study be exploited in case of other abiotic stress also. Besides, the interplay between ethylene and other phytohormones needs to be elucidated for As tolerance.

## Data Availability Statement

The datasets presented in this study can be found in online repositories. The names of the repository/repositories and accession number(s) can be found in the article/Supplementary Materials.

## Author Contributions

NK designed and supervised the experimentation and thoroughly edited the entire manuscript. ZS performed the experiments and generated the data. BR, MF, and NI analyze the data. ZS and MF jointly wrote the manuscript. MA reviewed and edited the manuscript. All authors contributed to the article and approved the submitted version.

## Conflict of Interest

The authors declare that the research was conducted in the absence of any commercial or financial relationships that could be construed as a potential conflict of interest.

## Publisher's Note

All claims expressed in this article are solely those of the authors and do not necessarily represent those of their affiliated organizations, or those of the publisher, the editors and the reviewers. Any product that may be evaluated in this article, or claim that may be made by its manufacturer, is not guaranteed or endorsed by the publisher.
