# Supplementary material for: Ethylene Suppresses Abscisic Acid, Modulates Antioxidant System to Counteract Arsenic-Inhibited Photosynthetic Performance in the Presence of Selenium in Mustard
Source: Front Plant Sci. 2022 May 16;13:852704. doi: 10.3389/fpls.2022.852704 (PMC9149584; doi:10.3389/fpls.2022.852704)
Supplement: Supplementary file 1 [file Table_1.DOC]

Supplementary Material

**Determination of H_2_O_2_ content**

The method of Okuda et al. (1991) was used for H_2_O_2_ determination. For this, 500 mg fresh leaves were macerated in ice-cold 200 mM HClO_4_ (perchloric acid). Thus, the obtained homogenate was subjected to centrifugation at 1500 × *g* for 10 min. The HClO_4_ of the supernatant was neutralized with 4 M KOH. The insoluble KClO_4_ was removed by further centrifugation at 500 × *g* for 3 min. The reaction mixture consisted of 1.5 ml eluate, 400 µL of 12.5 mM 3- dimethyl aminobenzoic acid (DMAB) in 0.375 M phosphate buffer (pH 6.5), 80 μL of 3-methyl-2- benzothiazoline hydrazone and 20 µL of peroxidase (0.25 unit). Peroxidase was added to start the reaction and increase in absorbance was recorded at 590 nm.

The content of thiobarbituric acid reactive substances (TBARS) was taken as the measure of lipid peroxidation and was estimated by the method of Dhindsa et al. (1981). The homogenization medium for 500 mg fresh leaves contained 0.25% 2- thiobarbituric acid in 10% trichloroacetic acid. The mixture was heated at 95^o^C for 30 min and then was rapidly cooled on ice bath, followed by centrifugation at 10,000 x *g* for 10 min. In 1.0 mL aliquot, 4.0 mL of 20% trichloroacetic acid containing 5% thiobarbituric acid was added. Finally, the color intensity was recorded at 532 nm.

**Assay of antioxidant enzymes activities**

The extraction buffer containing 0.05% (v/v) Triton X-100 and 1% (w/v) PVP in potassium-phosphate buffer (100 mM, pH 7.0) was used to homogenize 200 mg leaves in chilled mortar and pestle. The centrifugation was done at 15,000 x *g* for 20 min at 4^o^C. The supernatant thus collected was used for the assay of SOD (EC; 1.15.1.1) and GR (EC; 1.6.4.2) enzymes. The addition of 2.0 mM ascorbate with the extraction buffer was made for the assay of APX (EC; 1.11.1.11).

The methods given by Beyer and Fridovich (1987) and Giannopolitis and Ries (1977) were used SOD assay, which includes study of the inhibition of photochemical reduction of NBT. The 5.0 mL reaction mixture consisted of 5 mM HEPES (pH 7.6), 50 mM Na_2_CO_3_ (pH 10.0), 0.1 mM EDTA, 0.025% (v/v) Triton X-100, 13 mM methionine, 63 mmol NBT and 1.3 mmol of riboflavin. The enzyme extract was illuminated for 15 min (360 μmol m^2^ s^-1^), and a control set was not illuminated. The amount of enzyme that inhibited the reduction of NBT by 50% at 560 nm is equal to one unit of SOD.

For determining APX activity by the method of Nakano and Asada (1981) the decrease in the absorbance of ascorbate at 290 nm was recorded. A 1.0 mL assay mixture consisted of 50 mM phosphate buffer (pH 7.0), 0.1 mM EDTA, 0.5 mM ascorbate and 0.1 mM H_2_O_2_, and the enzyme extract. APX activity was calculated by using the extinction coefficient of 2.8 mM^-1^cm^-1^. One unit of the enzyme is the amount necessary to decompose 1 µmol of substrate per min at 25^o^C.

The activity of GR was determined by monitoring the glutathione-dependent oxidation of NADPH at 340 nm as given by Foyer and Halliwell (1976). The reaction mixture was phosphate buffer (25 mM, pH 7.8), 0.5 mM GSSG, 0.2mM NADPH, and the enzyme extract. The calculation of GR activity was done using the extinction coefficient 6.2 mM^-1^ cm^-1^. One unit of enzyme is the amount necessary to decompose 1 µmol of NADPH per min at 25^o^C.

**Determination of reduced glutathione content**

GSH content was determined by Anderson (1985). The homogenization of fresh leaf tissue (0.5 g) was done in 2.0 mL of 5% sulphosalicylic acid at 4 °C at 10,000 × *g* for 10 min. The supernatant (0.5 mL) was taken and 0.6 mL of phosphate buffer (100 mM, pH7.0) and 40 mL of 5/ 5/-dithiobis-2-nitro benzoic acid (DTNB) were added. After two minutes, the absorbance was recorded at 412 nm.

**Measurement of ACS activity and ethylene evolution**

The methods of Avni et al. (1994) and Woeste et al. (1999) were used for 1-aminocyclopropane-carboxylic acid-synthase (ACS; EC, 4.4.1.14) activity measurement. The homogenized preparation from leaf tissue (5.0 g) grounded in 100 mM HEPES buffer (pH8.0) containing 4 mM DTT, 2.5 mM pyridoxal phosphate, and 25% PVP was centrifuged at 12,000 x *g* for 15 min. In a 30 mL tube 1.0 mL of the supernatant and 0.1 mL of 5 mM S-adenosyl methionine (AdoMet) were added, and incubated for 2 h at 22ºC. In the reaction, the ACC formed was determined by its conversion to ethylene by the addition of 0.1 mL of 20 mM HgCl_2_ followed by the addition of 0.1 mL of a 1:1 mixture of saturated NaOH/NaCl and placed on ice for 10 min. In the control set, AdoMet was not added.

Evolution of ethylene was measured by cutting 500 mg of plant leaf into small pieces and placed into 30 ml tubes containing moist paper for minimizing the evaporation from the tissues and stoppered with secure rubber caps and kept in light for 2 h under the same condition used for the plant growth. An earlier experiment showed that 2 h of incubation time was appropriate for ethylene detection without the interference of wound induced ethylene, which started after 2 h of leaf incubation. A 1 mL of gas samples from the tubes were taken by a hypodermic syringe and assayed on a gas chromatograph (Nucon 5700, New Delhi, India) endowed with a 1.8 m porapack N (80-100 mesh) column, a flame ionization detector and data station. Nitrogen was used as the carrier gas. The flow rates of hydrogen, nitrogen and oxygen were 30, 30 and 300 mL min^-1^ respectively; the detector was set at 150^o^C. Ethylene was detected based on retention time and measured by comparison with peaks from standard ethylene concentration.

**Abscisic acid determination**

The content of ABA was determined by adopting the method of Hung and Kao (2003) with slight modiﬁcations. Leaves were frozen with liquid nitrogen immediately and ground into ﬁne powder. The powder was homogenized in the extraction solution (80% methanol containing 2% glacial acetic acid). The crude extract was centrifuged and passed through polyvinylpyrrolidone column and C18 cartridges to remove plant pigments and other non-polar compounds which could interfere in the immunoassay. The eluates were then concentrated to dryness by vacuum evaporation and resuspended in Tris-buﬀered saline before enzyme-linked immunosorbent assay (ELISA). Afterwards, ABA was determined with ABA immunoassay detection kit (PGR-1; Sigma-Aldrich, St. Louis, MO, USA) as per the user manual. The ABA content was estimated from a calibration curve plotted by using standard ABA and values were recorded at 405 nm.

**Histochemical staining method**

Histochemical staining was performed using the protocol provided by Kumar et al. (2014). Nitro blue tetrazolium (NBT) and 3, 3’-Diaminobenzidine (DAB) were used for the assay of accumulation the superoxide ion O_2_^-^ and H_2_O_2_ in samples. The samples from each treatment were kept into NBT solution prepared by dissolving 0.1 g NBT in 50 ml of 50mM sodium phosphate buffer (pH 7.5) in an amber coloured bottle and were kept for overnight. The stained samples were immersed in absolute ethanol and boiled in water-bath for 10 min for discoloration to get the staining clear.

For DAB staining solution, 50 mg DAB dissolved in 50 mL double distilled water in an amber coloured bottle with pH to 3.8. The samples from each treatment were kept into DAB solution and incubated it for 8 hours. The stained samples were immersed in absolute ethanol and boiled in water-bath for 10 min for discoloration to visualize the staining clear.

**Rubisco activity**

The activity of Rubisco was determined by monitoring NADH oxidation at 30^o^C at 340 nm when 3- phosphoglycerate is converted into glycerol-3-phosphate after addition of enzyme extract to the reaction mixture (Usuda, 1985). Leaf tissue (1.0 g) were homogenized in a chilled mortar and pestle with ice-cold extraction buffer containing 0.25 M Tris-HCl (pH 7.8), 0.0025 mM EDTA, 0.05 mM MgCl_2_, and 37.5 mg DTT for enzyme extraction. The homogenate was centrifuged at 10,000× g for 10 min at 4^o^C. The supernatant obtained after centrifugation was used to assay the enzyme. The reaction mixture contained 100 mM Tris-HCl (pH8.0), 10 mM MgCl_2_, 40 mM NaHCO_3_, 4.0 mM ATP, 0.2 mM NADH, 5.0 mM DTT, 0.2 mM EDTA, 1.0 U of glyceraldehydes-3-phosphodehydrogenase and 1.0 U of 3-phosphoglycerate-kinase and 0.2 mM of ribulose1,5-bisphosphate.

**Table S1.** Primer sequences of genes used for quantitative RT-PCR

| **S.No.** | **NCBI accession No.** | **Name of the encoding protein** | **Gene name** | **Primer sequence** |
| --- | --- | --- | --- | --- |
| **1** | AF038839 | Ascorbate oxidase | **APX** | F-ACCTGACATTCCTTTCCACC  R- AACCATCTGCTTTAGTAACACC |
| **2** | AF109694 | Glutathione reductase | **GR1** | F-TACTACTCCTCCTCCTCCTC  R- CACATCCTCTAAGCACACAC |
| **3** | HM565958 | ACTIN | **Actin** | F-TGAGCAAGGAAATCACGGC  R-GGACAATGGATGGACCTGAC |

**References**

Anderson, M. E. (1985). Determination of glutathione and glutathione disulfide in biological samples. *Meth. Enzymol.* 113, 548-555. https://doi.org/10.1016/j.jchromb.2009.06.016

Avni, A., Bailey, B. A., Mattoo, A. K., and Anderson, J. D. (1994). Induction of ethylene biosynthesis in *Nicotiana tabacum* by a Trichoderma-viride- xylanase is correlated to the accumulation of 1-aminocyclopropane-1-carboxylic acid (ACC) synthase and ACC oxidase transcripts. *Plant Physiol.* 106, 1049-1055. https://dx.doi.org/10.1104%2Fpp.106.3.1049

Beyer, Jr., W.F., and Fridovich, I. (1987). Assaying for superoxide dismutase activity: some large consequences of minor changes in conditions. *Anal. Biochem*. 161, 559-566. https://doi.org/10.1016/0003-2697(87)90489-1

Dhindsa, R. S., Plumb-Dhindsa, P., and Thorpe, T. A. (1981). Leaf Senescence: Correlated with increased levels of membrane permeability and lipid peroxidation, and decreased levels of superoxide dismutase and catalase. *J. Exp. Bot.* 32, 93-101. https://doi.org/10.1093/jxb/32.1.93

Foyer, C.H., and Halliwell, B. (1976). The presence of glutathione and glutathione reductase in chloroplasts: a proposed role in ascorbic acid metabolism. *Planta* 133, 21-25. <https://www.jstor.org/stable/23372114>

Giannopolitis, C.N., and Ries, S.K. (1977). Superoxide dismutases: I. Occurrence in higher plants. *Plant Physiol*. 59, 309-314. https://doi.org/10.1104/pp.59.2.309

Hung, K. T., and Kao, C. H. (2003). Nitric oxide counteracts the senescence of rice leaves induced by abscisic acid. *J. Plant Physiol.* 160, 871-879. https://doi.org/10.1078/0176-1617-01118

Kumar, D., Yusuf, M.A., Singh, P., Sardar, M., and Sarin, N.B. (2014). Histochemical detection of superoxide and H_2_O_2_ accumulation in Brassica juncea seedlings. *Bio-Protoc*. 4, e1108-e1108. https://doi.org/10.21769/BIOPROTOC.1108

Nakano, Y., and Asada, K. (1981). Hydrogen peroxide is scavenged by ascorbate-specific peroxidase in spinach chloroplast. *Plant Cell Physiol*. 22, 867-880. https://doi.org/10.1093/oxfordjournals.pcp.a076232

Okuda, T., Matsuda, Y., Yamanaka, A., and Sagisaka, S. (1991). Abrupt increase in the level of hydrogen peroxide in leaves of winter wheat is caused by cold treatment. *Plant Physiol.* 97, 1265-1267. https://doi.org/10.1104/pp.97.3.1265

Usuda, H. (1985). The activation state of ribulose 1, 5-bisphosphate carboxylase in maize leaves in dark and light. *Plant Cell Physiol*. 26, 1455-1463. https://doi.org/10.1007/BF00018268

Woeste, K. E., Ye, C., and Kieber, J. J. (1999). Two Arabidopsis mutants that overproduce ethylene are affected in the posttranscriptional regulation of 1-aminocyclopropane-1-carboxylic acid synthase. *Plant Physiol.*119, 521-530. https://doi.org/10.1104/pp.119.2.521
